# Novel Stopping Criteria for Optimization-Based Microwave Breast Imaging Algorithms

**DOI:** 10.3390/jimaging5050055

**Published:** 2019-05-22

**Authors:** Cameron Kaye, Ian Jeffrey, Joe LoVetri

**Affiliations:** Electrical and Computer Engineering, University of Manitoba, Winnipeg, MB R3T 5V6, Canada

**Keywords:** breast imaging, microwave imaging, discontinuous Galerkin method (DGM), contrast source inversion (CSI), stopping criteria, Kolmogorov-Smirnov (K-S) test

## Abstract

A discontinuous Galerkin formulation of the Contrast Source Inversion algorithm (DGM-CSI) for microwave breast imaging employing a frequency-cycling reconstruction technique has been modified here to include a set of automated stopping criteria that determine a suitable time to shift imaging frequencies and to globally terminate the reconstruction. Recent studies have explored the use of tissue-dependent geometrical mapping of the well-reconstructed real part to its imaginary part as initial guesses during consecutive frequency hops. This practice was shown to improve resulting 2D images of the dielectric properties of synthetic breast models, but a fixed number of iterations was used to halt DGM-CSI inversions arbitrarily. Herein, a new set of stopping conditions is introduced based on an intelligent statistical analysis of a window of past iterations of data error using the two-sample Kolmogorov-Smirnov (K-S) test. This non-parametric goodness-of-fit test establishes a pattern in the data error distribution, indicating an appropriate time to shift frequencies, or terminate the algorithm. The proposed stopping criteria are shown to improve the efficiency of DGM-CSI while yielding images of equivalent quality to assigning an often liberally overestimated number of iterations per reconstruction.

## 1. Introduction

Microwave imaging (MWI) has made steady progress towards widespread clinical application in breast cancer detection and monitoring over the past decade, offering a safety advantage over established x-ray-based modalities due to its use of non-ionizing radiation and a significant cost benefit over magnetic resonance imaging (MRI). While its currently attainable spatial resolution does not match that of cancer screening tools like mammography, concurrent use of MWI of the breast in its current state could lead to decreased false positive rates among certain population groups, particularly pregnant women or those with radiographically dense (“Category C” and “Category D”) breasts, as classified by the American College of Radiology’s Breast Imaging Reporting and Data System (BI-RADS) [1,2].

Methods of utilizing MWI for biomedical applications have been investigated for almost half a century [3,4]. Some recent representative research can be found in [5]. MWI algorithms can be split into those producing qualitative images, where the reconstructed value at each pixel is a relative quantity, and those producing quantitative reconstructions of a physical property, typically the complex-valued permittivity. Examples of qualitative algorithms are those utilized in so-called radar imaging techniques [6], and those retrieving only qualitative aspects of the target, such as its support [7,8]. The interest in this paper are quantitative imaging methods that solve the full electromagnetic inverse problem.

It is well known that the electromagnetic inverse problem associated with MWI, where one attempts to reconstruct the complex-valued permittivity of an object-of-interest (OI), is ill-posed [9]. The ill-posedness stems from the non-uniqueness of the inverse-source problem where one attempts to reconstruct the electromagnetic sources responsible for a remotely measured field. In the electromagnetic inverse problem, these sources are the contrast-sources that are illuminated by the interrogating field. Various regularization techniques have been developed over several decades of research to deal with the ill-posedness of the problem [10]. The electromagnetic inverse problem is not only ill-posed but also non-linear with repect to the two unknowns within the inaccessible imaging domain, the electromagnetic field and the permittivity. The Contrast Source Inversion (CSI) technique effectively linearizes the problem by casting the data-error norm in terms of contrast-source variables, which vary with the interrogating field, and regularization is performed by introducing the so-called Maxwellian regularizer which is written in terms of the contrast-sources and the contrast [11]. Much research has been performed on advancing the CSI algorithm since it was first reported, including the addition of a multiplicative regularizer [12].

For biomedical imaging applications, which are of interest in the context of the present work, the incorporation of discretized numerical inversion models into CSI that are based on either the finite-element method (FEM) or the discontinuous Galerkin method (DGM) forward solvers have provided much flexiblity and several advantages for the overall inversion process [13,14,15]. Most importantly, these forward solvers have allowed one to incorporate prior information into the inversion model that has the effect of regularizing the inversion, enabling high-contrast reconstructions such as are required for breast imaging [16,17,18,19]. The flexibility of these partial differential equation (PDE) based solvers has also allowed advancements in data-acquisition systems utilized to acquire scattered-field data for MWI [20,21]. Other potential advancements in MWI for breast tumor detection include the use of contrast agents [22].

Recent work in multi-frequency imaging has shown promise in increasing the spatial resolution and correspondingly, the amount of distinguishable anatomic detail in MWI reconstructions. For instance, while the use of “frequency hopping” has long been shown to be an effective means of obtaining images from high-frequency data by feeding low-frequency solutions as “initial guesses” into inversion algorithms [23], a so-called “frequency cycling” and “tissue-dependent mapping” framework has been adopted in recent studies to perform multi-frequency imaging demonstrating significant enhancement in the quality of resulting 2D images [24,25]. In those works, an arbitrary fixed number of iterations was used as the stopping condition for the imaging algorithm, but it was noted that further exploration into more intelligent stopping criteria was justified, particularly based on the observation that running the algorithm longer than necessary could deteriorate the imaging results. In fact, to date, there has been little exploration into optimizing the conditions to which a microwave imaging algorithm should adhere when determining the appropriate point to move on from intermediate inversions to the next frequency in a multi-frequency sequence, or when to terminate reconstructions entirely.

While monitoring the convergence of data error has been the most common objective approach to halting microwave imaging algorithms, as it is often difficult to ascertain whether a chosen error limit will undershoot, overshoot, or consistently meet the attainable image reconstruction quality for a given dataset, several early studies focusing on synthetic and experimental imaging performance fell into the same habit of simply pre-assigning an arbitrary number of iterations for the algorithm to complete, chosen for convenience or through trial and error [11,26,27,28].

Although there have been previous attempts to develop more intelligent stopping criteria for imaging algorithms in recent literature [17], a more thorough statistical analysis of the data error is undertaken here and employed as part of a logical multi-variable framework of stopping criteria for a frequency-cycling tissue-dependent mapping formulation of Contrast Source Inversion (CSI). For comparison purposes, some 2D imaging results from a recent study are included in this work to demonstrate the significant gains in efficiency and image quality granted through the incorporation of the described stopping criteria [25]. Reconstruction results are subjected to quantitative analysis using common image error metrics to provide an objective measurement of these improvements.

## 2. Materials and Methods

### 2.1. DGM-CSI Algorithm

An implementation of CSI using a high-order frequency-domain formulation of Maxwell’s curl equations has been used for all 2D breast images presented here, employing the discontinuous Galerkin method (DGM) as a forward solver. Nodal coefficients in high-order polynomial expansions represent unknown field and contrast quantities in the resulting DGM-CSI algorithm. Independent from this choice of implementation, among the benefits of CSI is its use of operators that are functions of frequency and the material properties of the background medium only, which do not change from iteration to iteration [11]. As aforementioned, further improvement in performance has been demonstrated through the use of a tissue-dependent mapping process and the practice of cycling back to low-frequency reconstructions [25], and a brief overview of this process is provided in Section 2.2. Further details specific to DGM-CSI can be found in [14,29], but a summary of the relevant quantities and definitions involved in the CSI cost functional is included here, since it pertains to the discussion of data and domain error that follows.

**Figure 1:**
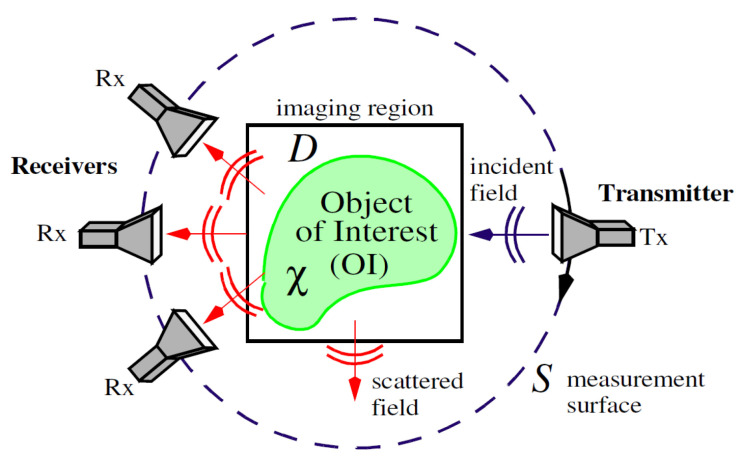
Two-dimensional representation of the imaging problem. The object of interest (OI) has an unknown contrast χ, where D is the imaging domain and S is the surface containing the transmitters (Tx) and receivers (Rx).

The microwave imaging problem in Figure 1 depicts an object of interest (OI) with an unknown dielectric contrast $\chi (\vec{r})$, a function of position vector $\vec{r}$, typically defined as

(1) $$\chi \left(\vec{r}\right)=\frac{{\hat{\varepsilon }}_{r}\left(\vec{r}\right)-{\hat{\varepsilon }}_{b}}{{\hat{\varepsilon }}_{b}},$$

where ${\hat{\varepsilon }}_{r}$ is the complex relative permittivity and ${\hat{\varepsilon }}_{b}$ is the complex background permittivity. In breast imaging applications, the background permittivity is usually represented as the homogeneous immersion medium in which the breast is submerged and the imaging domain D is an area typically chosen to be contained by the outer skin layer of the breast. An array of t=1,2,…,T transmitters is used to generate probing incident fields; the resulting scattered fields are measured by an array of *R* receivers that make up a discrete measurement surface S.

It is assumed that the reader is familiar with the standard CSI algorithm, and only the aspects important to the proposed stopping criteria will be reviewed. The CSI algorithm solves for the contrast $\chi (\vec{r})$ of breast tissue within D from this scattered field data by minimizing the following two-part cost functional:

(2) $$F^{\mathrm{CSI}}\left(\chi ,w_{t}\right)=F^{S}\left(w_{t}\right)+F^{D}\left(\chi ,w_{t}\right),$$

where both the contrast $\chi (\vec{r})$ and the transmitter-dependent contrast sources $w_{t}\left(\vec{r}\right)=\chi \left(\vec{r}\right)u_{t}^{\mathrm{tot}}$ (a product of the contrast and the total field $u_{t}^{\mathrm{tot}}$) are unknown and confined to the domain D.

The first term of Equation (2) represents the “data error”, given by

(3) $$F^{S}\left(w_{t}\right)=\frac{\sum_{t}{∥u_{t}^{\mathrm{sct}}-L_{S}\left\{w_{t}\right\}∥}_{S}^{2}}{\sum_{t}{∥u_{t}^{\mathrm{sct}}∥}_{S}^{2}},$$

and correspondingly, the second term represents the “domain error”,

(4) $$F^{D}\left(\chi ,w_{t}\right)=\frac{\sum_{t}{∥\chi u_{t}^{\mathrm{inc}}-w_{t}+\chi L_{D}\left\{w_{t}\right\}∥}_{D}^{2}}{\sum_{t}{∥\chi u_{t}^{\mathrm{inc}}∥}_{D}^{2}},$$

with ${∥\cdot ∥}_{D}$ and ${∥\cdot ∥}_{S}$ denoting the $L_{2}$ norms on D and S. The measured scattered field data is represented above by $u_{t}^{\mathrm{sct}}$ at *R* receiver locations per transmitter *t*, and the incident field inside D is $u_{t}^{\mathrm{inc}}$. The forward operator $L_{S}$ converts contrast source estimates $w_{t}$ in D to scattered field values at *R* receiver points per transmitter *t* on the measurement surface S; $L_{D}$ performs a similar function, but transforms contrast sources to scattered field values within D. For further simplicity, the “data error” per iteration *i* can be defined from the numerator of Equation (3) as

(5) $$\rho_{t}^{\left(i\right)}=d_{t}-L_{S}\left\{w_{t}^{\left(i\right)}\right\}$$

and correspondingly, the “domain error” from the numerator of Equation (4) as

(6) $$r_{t}^{\left(i\right)}=\chi^{\left(i\right)}u_{t}^{\mathrm{inc}}-w_{t}^{\left(i\right)}+\chi^{\left(i\right)}L_{D}\left\{w_{t}^{\left(i\right)}\right\}.$$

These error values, particularly the data error of Equation (5), are subject to analysis for evaluating convergence behaviour of DGM-CSI in this study, described in Section 2.3.

### 2.2. Frequency-Cycling Tissue-Dependent Mapping Technique

The well-documented dielectric properties of breast tissues lead to the observation that the geometry of the real and imaginary parts of tissues’ permittivity profiles should be qualitatively similar [24,25]. The quantitative permittivity values of different types of breast tissue also form reasonably discrete ranges that are highly correlated between real and imaginary components, making it easy to stratify these values into expected tissue types (i.e., fat, transitional, fibroglandular, and cancer) [30]. Through a tissue-dependent mapping of the imaginary part based on geometric and quantitative reconstructions of the dielectric constant, an “artificial” imaginary component was proposed as part of the initial guess for the next frequency dataset in the imaging sequence, to good effect [24,25].

In addition to tissue-dependent mapping, this previous work invoked “frequency cycling” as opposed to conventional frequency hopping, where frequency cycling simply continues the reconstruction process past the highest frequency in a multi-frequency sequence by returning to the lowest frequency dataset (with an initial guess based on the highest frequency solution) [25]. The initial studies using DGM-CSI have shown that this practice preserves the fine detail acquired from high-frequency data, and produces better results than a single incremental frequency-hopping sequence operating for the same total number of iterations. In a practical microwave imaging scenario without the benefit of foreknowledge of this total number of iterations, however, a potential drawback to frequency cycling would be the increased number of iterations required to produce images when arbitrary or overly-stringent stopping conditions are applied.

### 2.3. Stopping Criteria for Single-Frequency Reconstructions

As described in Section 2.1, iterative inversion methods such as DGM-CSI attempt to solve the inverse scattering problem by minimizing the cost functional of Equation (2), which includes a data error $\rho_{t}^{\left(i\right)}$ (Equation (5)) represented by the difference between the measured and computed fields on S for a given transmitter *t*. If *U* is the total number of data points across all receivers *R* and sources *T*, then the U×1 data-error vector ${\bar{\rho }}^{\left(i\right)}$ can be represented as a concatenation of every transmitter’s data errors, such that

(7) $${\bar{\rho }}^{\left(i\right)}=\left[\rho_{1}^{\left(i\right)};\rho_{2}^{\left(i\right)};\ldots ;\rho_{T}^{\left(i\right)}\right]$$

After all contrast sources $w_{t}^{\left(i\right)}$ are updated for each transmitter *t*, the inversion process by convention performs a convergence check that calculates the new data-error vector ${\bar{\rho }}^{\left(i+1\right)}$. The goal is to reduce this new data error as much as possible, and aside from simply assigning a maximum number of iterations, one could monitor the data error until it falls below an arbitrarily-chosen tolerance level δ such that some form of a termination condition examining its sum, maximum value, or norm applies, commonly implemented as

$$\mathrm{if}\;(∥{\bar{\rho }}^{\left(i+1\right)}∥<\delta )\;\mathrm{stop}.$$

Alternatively, the difference between the normalized data-error vector at two successive iterations, ${\bar{\rho }}^{\left(i\right)}$ and ${\bar{\rho }}^{\left(i+1\right)}$, can be calculated until it falls within a prescribed tolerance to check for convergence [26].

Such conditions would seem to be reasonable criteria by which termination of the inversion algorithm should take place. However, the total data error often has little bearing on the accuracy of the resulting reconstruction, despite properly converging to a preset minimum. While liberally large values of error tolerance will logically terminate the imaging algorithm prematurely, it has also been observed that conservatively small choices of error tolerance will result in a poorer quality image. This outcome is likely due to the supposition that the minimization of a cost functional towards zero arguably means accounting for any and all error in modeling, calibration, and measurements (i.e., noise) through non-physical changes in the reconstructions. The apparent inadequacy of having the data error converge to within an arbitrary tolerance alone served as motivation to develop a multifaceted approach to stopping criteria that improves the reconstructions of a frequency-cycling reconstruction technique through statistical analysis of data and domain error of each iteration.

This proposed framework stops the imaging algorithm based primarily on the statistical *distribution* of the contributing terms to the data-error vector $\bar{\rho }$. Referring the reader back to Equation (7), assuming a best-case scenario where the forward operator $L_{S}$ is able to perfectly model the field behaviour of the imaging system and the contrast sources $w_{t}$ had been iteratively solved to the exact solution, the data error $\rho_{t}$ would be reduced to the difference between the field values $d_{t}$ experimentally measured at the receivers for all given transmitters and the ideal noise-free field values generated by $L_{S}\left\{w_{t}^{\left(i\right)}\right\}$. This difference would amount to the noise of the experimental system, which would presumably converge to a known pattern, such as a Gaussian distribution.

However, it is clear that even if one could acquire completely noise-free measurement data, it is not practical, or even possible, to find a numerical model that could exactly match it. This so-called modeling error, attributed to mismatches between the approximate representation of the imaging environment $L_{S}$ (which varies by formulation, mesh granularity, order number, etc.) and the true, unknowable functions that perfectly predict field behaviours within the system, invalidates the assumption that the remaining data-error vector $\bar{\rho }$ (upon convergence of the imaging algorithm to a solution) consists simply of noise. Modeling error is the primary reason why data calibration must be performed on experimental data, and it is not a trivial issue to address in MWI [31].

Correspondingly, since the contributions to the data error are multifactorial in nature and vary with each experimental set-up and possibly with each target, it will not necessarily converge to a known family of statistical distributions. Use of any of the myriad of statistical normality tests available (such as the Anderson–Darling or Shapiro–Wilk test) on the data error as part of the proposed algorithmic stopping criteria would therefore be unreliable. However, the profile of the data error distribution (regardless of its final form) should reach a steady state when the algorithm has sufficiently converged to a particular solution. To confirm that this trend occurs during a reconstruction requires comparison of the current iteration’s data error distribution to those of multiple previous iterations, which is accomplished in this work with an implementation of the *two-sample Kolmogorov-Smirnov* (K-S) test.

The two-sample K-S test is a non-parametric goodness-of-fit hypothesis test that evaluates the maximum absolute difference *D* between the cumulative distribution functions (CDF) of two sample data vectors over a range of *x* in each data set. Recall that the CDF ($F_{X}$) of a continuous random variable *X* can be expressed as the integral of its probability density function (PDF) $f_{x}$ (that is, a function whose value at any given sample can be interpreted as providing a relative likelihood that the value of the random variable would be equal to that sample), such that

(8) $$F_{X}\left(x\right)=\int_{-\infty }^{x}f_{X}\left(t\right)\;dt.$$

In the discrete case, $F_{X}\left(x\right)$ would be equivalent to the proportion of values in $f_{X}\left(x\right)$ less than or equal to a specified value of *x*.

Suppose the data-error vector PDFs from two consecutive iterations of the DGM-CSI imaging algorithm, ${\bar{\rho }}^{\left(i\right)}$ and ${\bar{\rho }}^{\left(i+1\right)}$, each have some observed CDF, say ${\hat{P}}^{\left(i\right)}\left(x\right)$ and ${\hat{P}}^{\left(i+1\right)}\left(x\right)$; that is, for any specified value of *x*, the value of $\hat{P}\left(x\right)$ is the proportion of error values less than or equal to *x* in the corresponding dataset. Consider the quantity

(9) $$D=\max_{x}\left(\right|{\hat{P}}^{\left(i+1\right)}\left(x\right)-{\hat{P}}^{\left(i\right)}\left(x\right)\left|\right),$$

that represents the maximum error between two CDFs. The K-S test makes use of this *D* value to return a probability, in the form of a scalar asymptotic *p*-value in the range of [0,1], which represents the likelihood that the two samples were drawn from the same distribution. More precisely, the *p*-value is defined as the probability of observing a test statistic (*D*) as extreme or more extreme than the observed value, for the null hypothesis that the data in these two vectors are from the same continuous distribution [32,33,34,35]. In the implementation of the test, the decision to reject the null hypothesis is based on comparing the returned *p*-value with a preset significance level (commonly 5% for many applications). The test becomes very accurate for large sample sizes, and is believed to be reasonably accurate for sample sizes $U_{1}$ and $U_{2}$, such that $\left(U_{1}\times U_{2}\right)/\left(U_{1}+U_{2}\right)\geq 4$, which is the case for a collection of data error values in a system of 24 transmitters and receivers operating at a single frequency (representing a total of 576 data points).

While the strength of the two-sample K-S test lies in determining when two samples are from differing distributions, the nature of the test guarantees that the smaller the value of *D*, the higher the returned *p*-value will be. This feature allows the test’s *p*-value to be used as a numerical indicator for how likely the two samples were drawn from the same distribution (i.e., how closely their CDFs match), as opposed to its primary use in *rejecting* the null hypothesis.

**Figure 2:**
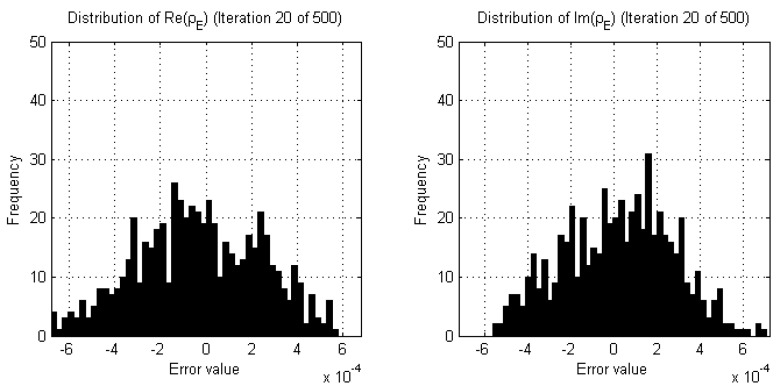
Examples of histograms demonstrating estimates of the probability distribution of the real and imaginary parts of the data error ($\rho_{E}$) during a DGM-CSI inversion of an arbitrary 2D synthetic breast model early in the reconstruction process (iteration 20).

**Figure 3:**
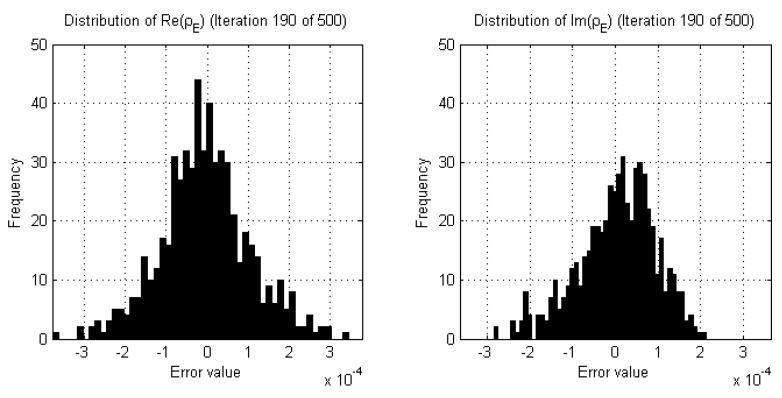
Examples of histograms demonstrating estimates of the probability distribution of the real and imaginary parts of the data error ($\rho_{E}$) during the same DGM-CSI inversion as Figure 2, later in the reconstruction process (iteration 190).

Empirical studies of the real and imaginary parts of the data error of DGM-CSI reconstructions indeed revealed that the shape of the probability distribution curves fluctuate significantly with large variances in early iterations (Figure 2) but gradually reach a steady state in later iterations as expected, once all remarkable subjective change in the resulting images had ceased (Figure 3). Since any two neighboring iterations’ data error may only change slightly and be interpreted as being drawn from the same distribution by the two-sample K-S test, it was decided that a sliding window of past iterations’ data errors should be examined and compared to that of the current iteration. Moreover, the threshold value that the returned *p*-value should surpass for any given K-S test within this sliding window needed to be high enough to similarly reject subtler variations in two iterations of data error that would otherwise pass the goodness-of-fit test. This wider breadth of analysis ensured that more gradual changes were detected so that any corresponding stopping criteria would not halt the reconstruction prematurely.

Therefore, the window size of iterations under scrutiny, the *p*-value threshold (assumed constant over the window), and the percentage of that window’s iterations reaching or surpassing that *p*-value threshold, are all parameters to the stopping criteria for the algorithm based on the K-S test, which is applied separately for the real and imaginary parts of each iteration’s data error. A preliminary study attempting to establish the effects of these parameters was undertaken for the frequency-cycled reconstruction of a 2D breast model, described in Section 3.1.

It should be noted that the statistical analysis governing the aforementioned stopping criteria only makes use of the *data* error, which may be sufficient for certain MWI algorithms (such as the distorted Born iterative method) whose minimized cost functional consists solely of a data error term. However, as shown in Section 2.1, CSI-based iterative inversion algorithms make use of cost functionals containing a second domain error term dealing with total field values inside the imaging domain [11,26]. Therefore, another ad hoc stopping condition based on the domain error is also prudently employed in the algorithm, as described in the next subsection.

### 2.4. Global Termination of Multi-Frequency Reconstructions

The stopping conditions described in Section 2.3 are appropriate for halting single-frequency inversions, but multiple-frequency reconstructions have thus far not been addressed. In a frequency-hopping scenario, the stopping criteria based on K-S tests alone can be easily employed to determine when each individual inversion should terminate and move on to the next frequency, with global termination occurring following reconstruction of the highest frequency.

However, in a frequency-cycling reconstruction scheme, the cycle of reconstructions could theoretically continue *ad infinitum* without another explicit set of rules in place governing the global termination of the imaging process. A separate, secondary ad hoc global termination criterion was therefore implemented, based on a previous study [17]; once each frequency’s dataset in the cycle is subjected to the K-S test stopping criteria at least once, if the percentage of relative change in domain error between two successive iterations falls below 0.1%, then it is deemed appropriate to globally terminate the frequency cycle and end the reconstruction. This provision could also serve as a fallback contingency in the event that the data error-based conditions turn out to be too strict to satisfy for a given individual frequency.

### 2.5. Full Description of Multi-Frequency Imaging Procedure

The key concepts involved in the proposed multi-frequency imaging procedure, while implemented herein for the DGM-CSI algorithm, can easily be applied (with minor modifications) to other iterative algorithms widely used in MWI, including Gauss-Newton Inversion (GNI) and distorted Born Iterative Method (DBIM). As such, a descriptive approach to the technique is undertaken here in order to keep its applicability as general as possible. Taking into account the stopping criteria described throughout Section 2.3 and using the procedural description of the frequency-cycling reconstruction approach with tissue-dependent mapping described in [25], the new consolidated multi-frequency imaging procedure is proposed as follows:

1. The real and imaginary parts of the complex permittivity are reconstructed using the lowest frequency data available (e.g., 1.0 GHz). The termination point of this reconstruction is dictated by the results of successive two-sample K-S tests performed on both the real and imaginary parts of the data error separately, comparing the current iteration to those of a sliding window of past iterations, governed by a choice of parameters for *p*-value, window size, and percentage of windowed iterations reaching this *p*-value threshold. For robustness, a back-up termination condition may be implemented, either related to the relative change in domain error for CSI-based algorithms as described in Section 2.4, or a maximum number of iterations.
2. A point-by-point search through the reconstructed real part of each nodal basis coefficient in the DGM-CSI mesh (or more generally, each mesh element or pixel of the reconstructed image) classifies the type of breast tissue. This classification is based solely on the range of expected values of dielectric constant at that frequency, as outlined in [25].
3. An initial guess for the next imaging frequency (e.g., 2.0 GHz) is generated using the tissue-dependent mapping process [24,25]. It consists of the unmodified real parts of the reconstructed $\varepsilon_{r}$ at the mesh nodal points, and a new imaginary part created from a simple linear interpolation of the expected range of dielectric loss values, based on the appropriate Cole-Cole models of tissues classified in Step 2. This technique preserves the geometry of the real and imaginary parts of the solution.
4. This new initial guess for the complex permittivity is used to run the inversion algorithm at the next frequency (e.g., 2.0 GHz). As per the procedure outlined in [25], the user may choose to keep the imaginary part constant during this inversion and update only the real part to converge to a new solution. This “anchoring” process has been shown to improve overall imaging results due to the tendency of CSI-based inversion algorithms to cause significant deterioration of the imaginary part at high-frequency reconstructions. Again, the aforementioned parameterized stopping criteria would be primarily employed to determine the appropriate point to halt this reconstruction.
5. If more than two frequencies are used in the frequency hop, steps 2–4 are repeated as necessary until the reconstruction of the final frequency of the succession is complete (e.g., 3.0 GHz). This succession may include “frequency cycling”; that is, returning the inversion algorithm to the lowest frequency data and incrementally stepping through each frequency again [25].

If a simple frequency-hopping scheme is employed, the reconstruction will terminate after the highest-frequency inversion, again as decided by the parameterized stopping criteria based on K-S tests of the data error. However, if a frequency-cycling scheme is used, and if the imaginary part of the solution has been “anchored” in place following the first inversion in Step 1, there are further considerations that come into play for global termination of the imaging process, and to address concerns of full CSI optimization:

- When each available dataset in the frequency cycle has been used *at least once* to contribute to the overall image reconstruction, a global termination criterion will become active, which will monitor the relative change in the domain error between successive iterations (Section 2.4). If this relative change falls below 0.1% at any point, the current reconstruction is halted and the frequency cycle is broken.
- Regardless of the frequency at which the algorithm was halted by this relative domain error threshold, if the imaginary part of the solution has been continuously held constant during the frequency cycle after Step 1, one last initial guess is generated as in Step 3 and a final reconstruction is run at the lowest frequency available (e.g., 1.0 GHz) with both the real and imaginary parts allowed to converge to a solution (i.e., the imaginary part is no longer “anchored”). This final inversion is terminated by the parameterized stopping criteria *or* a relative change of domain error between successive iterations falling below 0.1%, whichever occurs first. The purpose of this final run is to demonstrate the stability of the final solution and ensure that its imaginary part, despite being originally based on the geometry and tissue properties of the real part, does indeed satisfy full CSI optimization.

### 2.6. Synthetic Breast Models

**Figure 4:**
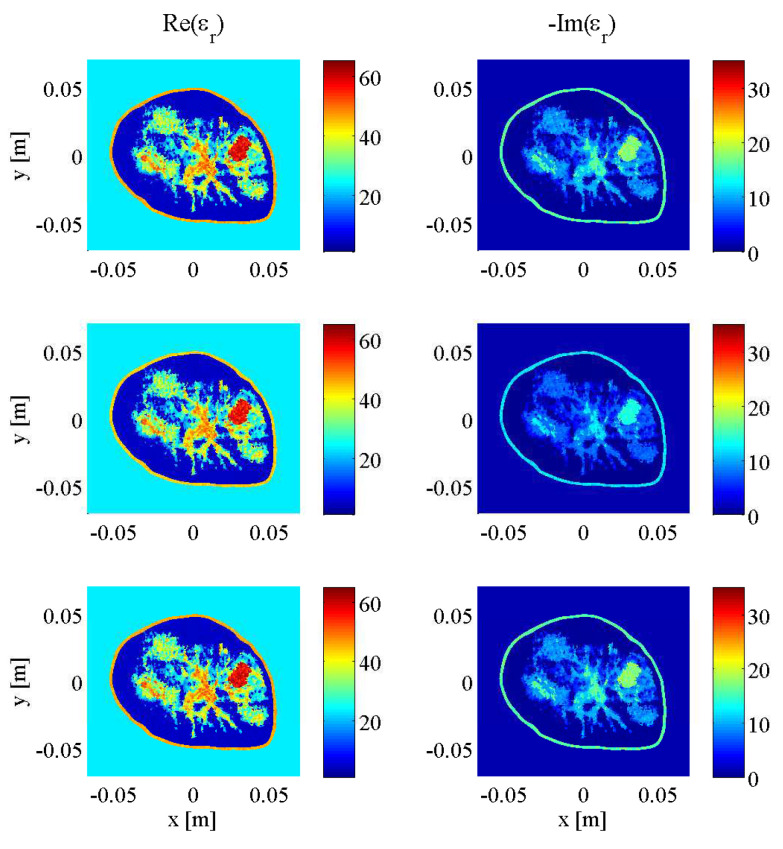
Complex dielectric properties of University of Calgary 2D synthetic breast model at 1.0 GHz (**top**), 2.0 GHz (**middle**) and 3.0 GHz (**bottom**).

**Figure 5:**
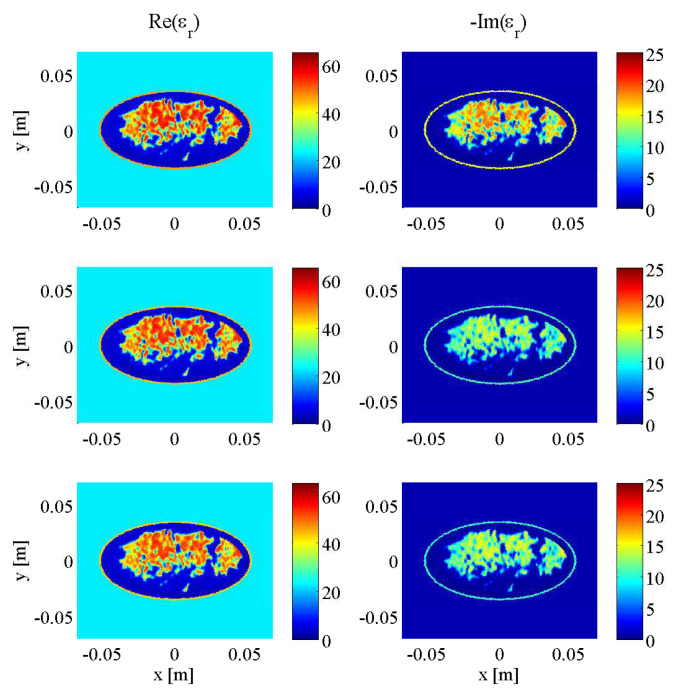
Complex dielectric properties of University of Wisconsin 2D synthetic breast model at 1.0 GHz (**top**), 2.0 GHz (**middle**) and 3.0 GHz (**bottom**).

Initial testing of the described stopping criteria and comparison to fixed-iteration scenarios were carried out on synthetic transverse magnetic (TM) data collected from a two-dimenstional MRI-derived BI-RADS Category C (heterogeneously dense) 2D breast model supplied by the University of Calgary, derived from an MRI slice of a cancer patient with a breast tumour visible at the “3 o’clock” position. To demonstrate the robustness of the technique across different breast phantoms, an additional two-dimensional MRI-derived BI-RADS Category D (“extremely dense”) healthy breast model supplied by the University of Wisconsin’s public database (ID: 070604PA2) was employed. Both models are depicted at three different frequencies (1.0 GHz, 2.0 GHz, and 3.0 GHz) in Figure 4 and Figure 5, illustrating the similarities between breast tissues’ dielectric permittivity at the low-gigahertz band. The values of the tissue-dependent complex permittivity in all cases were calculated for every frequency using an appropriate fitted single-pole Cole-Cole model (or equivalently, a single-pole Debye model as the exponent parameter α=0), and for the University of Calgary model, then subjected to random perturbations of ±10% [36].

Data was collected for both models in a low-loss background of $\epsilon_{r}=23-1.13i$ with 24 transmitters and 24 receivers evenly distributed at a radius of 10 cm, employing a finite-element method (FEM) forward solver with a finely-discretized mesh independent from that used for the DGM-CSI inversion. The synthetic data was collected for open boundary conditions at 1.0 GHz, 2.0 GHz, and 3.0 GHz. To test the robustness of the stopping criteria to problems with PEC boundaries, and to better reflect the capabilities of a recent University of Manitoba imaging system prototype [37], data was collected a second time with the Category C model surrounded by a circular PEC boundary with a radius of 15 cm at a reduced bandwidth. The frequencies used for the PEC-bounded case were 1.0 GHz, 1.25 GHz, and 1.5 GHz. Uniformly-distributed noise at 5% of the maximum field magnitude was used to corrupt the $E_{z}$ scattered electric field data for the Category C model, and several noise levels (3%, 5%, 7.5%, and 10%) were tested for the Category D model to evaluate the effect of noise on the stopping criteria’s performance. The skin thickness and inner skin boundary both remained unknown; the only prior information used during inversions was the outer skin boundary.

### 2.7. Error Calculation

An objective method of evaluating the DGM-CSI algorithm’s performance for differing imaging scenarios was accomplished by comparing the reconstruction results to the original Category C and Category D synthetic models of the *current* frequency being used in the reconstruction cycle, unless otherwise specified. The models and reconstructions each employed different underlying meshes, so their complex permittivity values were interpolated onto a common 250 × 250-pixel square grid (*N* = 62,500) that encompassed an area covering $\left(x_{1},y_{1}\right)=\left(-0.07,-0.07\right)$ [m] to $\left(x_{2},y_{2}\right)=\left(0.07,0.07\right)$ [m]. The relative error of $L_{1}$ and $L_{2}$ norms of the difference between the model (actual) complex permittivity ${\hat{\varepsilon }}_{r}^{\;\mathrm{act}}$ and the reconstructed complex permittivity ${\hat{\varepsilon }}_{r}^{\;\mathrm{rec}}$ over each pixel *k* were used as primitives to evaluate the performance of the imaging algorithm. For convenience, these will hereafter be referred to as the relative error norms ${\mathrm{REN}}_{1}$ and ${\mathrm{REN}}_{2}$, and are calculated and expressed as percentages, where

(10) $$\begin{array}{ccc} {\mathrm{REN}}_{1}\left({\hat{\varepsilon }}_{r}^{\;\mathrm{act}},{\hat{\varepsilon }}_{r}^{\;\mathrm{rec}}\right) & = & \frac{∥{\hat{\varepsilon }}_{r}^{\;\mathrm{act}}-{\hat{\varepsilon }}_{r}^{\;\mathrm{rec}}{∥}_{1}}{∥{\hat{\varepsilon }}_{r}^{\;\mathrm{act}}{∥}_{1}}\times 100\%\\ & = & \frac{\sum_{k=1}^{N}|{\hat{\varepsilon }}_{r}^{\;\mathrm{act}}\left(k\right)-{\hat{\varepsilon }}_{r}^{\;\mathrm{rec}}\left(k\right)|}{\sum_{k=1}^{N}|{\hat{\varepsilon }}_{r}^{\;\mathrm{act}}\left(k\right)|}\times 100\%, \end{array}$$

(11) $$\begin{array}{ccc} {\mathrm{REN}}_{2}\left({\hat{\varepsilon }}_{r}^{\;\mathrm{act}},{\hat{\varepsilon }}_{r}^{\;\mathrm{rec}}\right) & = & \frac{∥{\hat{\varepsilon }}_{r}^{\;\mathrm{act}}-{\hat{\varepsilon }}_{r}^{\;\mathrm{rec}}{∥}_{2}}{∥{\hat{\varepsilon }}_{r}^{\;\mathrm{act}}{∥}_{2}}\times 100\%\\ & = & \sqrt{\frac{\sum_{k=1}^{N}{\left[{\hat{\varepsilon }}_{r}^{\;\mathrm{act}}\left(k\right)-{\hat{\varepsilon }}_{r}^{\;\mathrm{rec}}\left(k\right)\right]}^{2}}{\sum_{k=1}^{N}{\left[{\hat{\varepsilon }}_{r}^{\;\mathrm{act}}\left(k\right)\right]}^{2}}}\times 100\%. \end{array}$$

The raw numerical value of the norms will be slightly reduced since the square grid covers an area *outside* of the imaging domain of the problem, which will consist of common unaltered background permittivity in both the model and reconstruction and thus represent pixels of zero error. Since the REN values serve primarily as a means of monitoring iterative trends in solution convergence and to demonstrate comparative improvement between imaging scenarios with equally-sized interpolated grids and imaging domains, the impact of this artificial error reduction is of little significance.

## 3. Results and Discussion

### 3.1. Imaging with Open Boundaries

**Figure 6:**
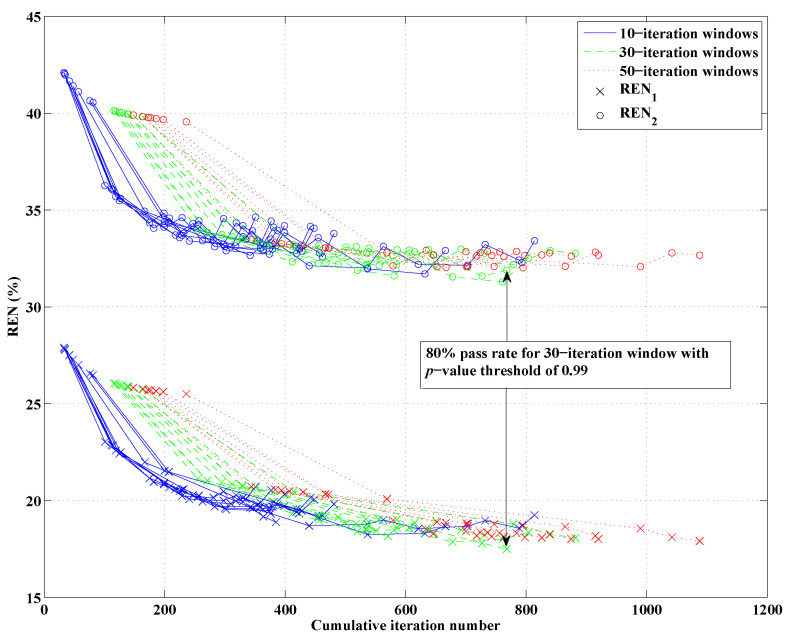
Relative error norms of open-boundary 2D DGM-CSI reconstructions across several choices of stopping criteria parameter values. Each curve in the figure corresponds to a frequency-cycled inversion for a particular choice of the three parameters (Section 3.1), with the window sizes coded by color for convenience. Data points on each curve correspond to REN values for the reconstructed model at each frequency change. Arrows point to the final REN values of simulations that terminated with the lowest relative error norms, indicating the best combination of parameters among those tested for this imaging scenario.

As mentioned in the introduction of the stopping criteria in Section 2.3, the approach had three variables: the *p*-value threshold that any given two-sample K-S test would use to conclude the two data error samples were drawn from the same distribution, the window size of past iterations’ data errors compared to that of the current iteration, and the percentage of that window’s iterations that would need to to reach or surpass the chosen *p*-value threshold in order to halt the reconstruction at the current frequency, deemed the “pass percentage”. For the same Category C breast model in Figure 4, DGM-CSI was run in a frequency-cycled configuration using the rules described in Section 2.5 for every combination of *p*-value thresholds of 0.90, 0.95 and 0.99, window sizes of 10, 30, and 50, and pass percentages of 80%, 90%, and 100%. There were 27 simulations whose total number of iterations would vary based on the laxity or stringency of stopping criteria associated with low and high variable values respectively. Error norms (${\mathrm{REN}}_{1}$ and ${\mathrm{REN}}_{2}$) for each scenario were plotted for every frequency jump (Figure 6), and the best variable combination (resulting in the lowest error metric magnitudes upon termination) was found to be a *p*-value threshold of 0.99, a window size of 30, and a pass percentage of 80%. This combination of values was subsequently used for all future simulations employing the stopping criteria. Note that in Figure 6 and in all subsequent plots of REN values, the *x*-axis’ number of iterations represents the cumulative total throughout all frequency-hopping or frequency-cycling steps of the reconstruction.

**Figure 7:**
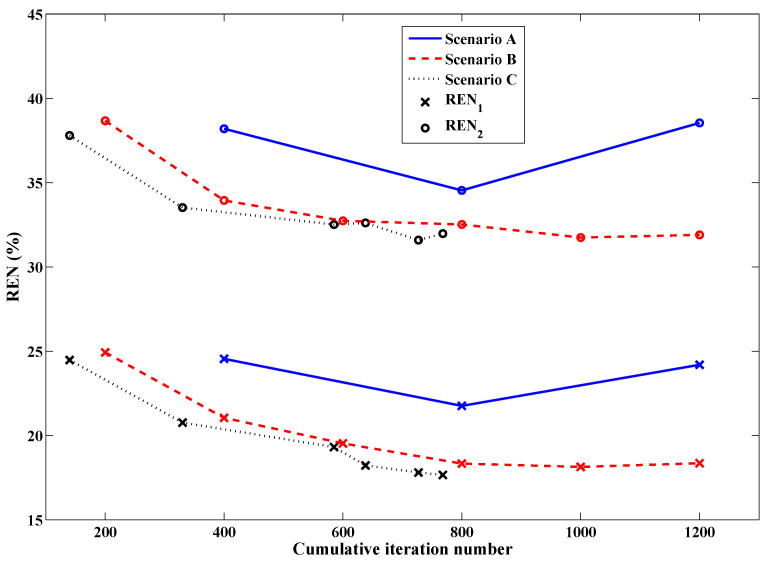
Relative error norms of frequency-hopping and frequency-cycled reconstructions: Scenario A (solid line)—without tissue-dependent mapping at 400 iterations per frequency terminating after first inversion of 3.0 GHz, Scenario B (dashed line)—with tissue-dependent mapping at 200 iterations per frequency, cycling through reconstruction frequencies once (with imaginary component “anchored” following initial 1.0 GHz inversion), Scenario C (dotted line)—with tissue-dependent mapping and stopping criteria in place, terminating after two consecutive inversions of 1.0 GHz data (one with the imaginary component “anchored” and the final run with the imaginary component freely optimized according to the guidelines of Section 2.5). See Table 1 for further details.

**Figure 8:**
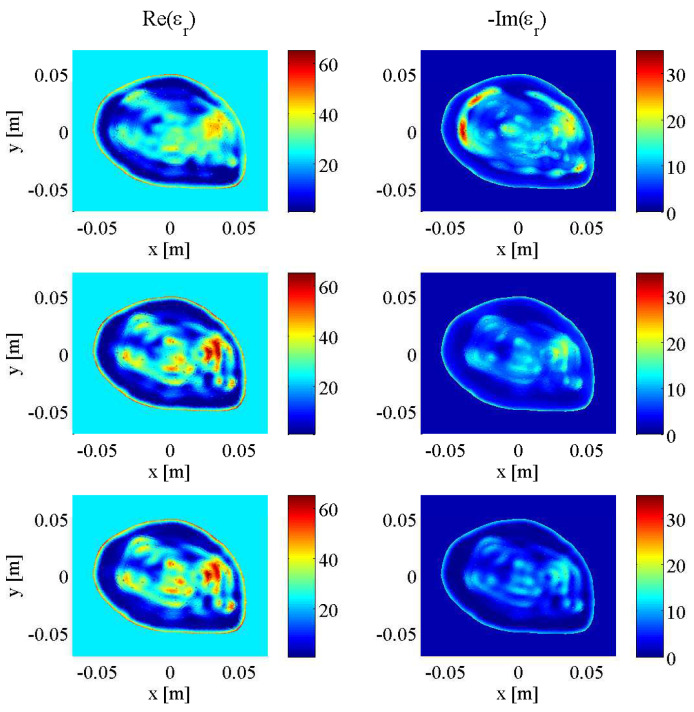
Final results of DGM-CSI frequency-hopping and frequency-cycled complex dielectric property reconstruction of synthetic breast model using 1.0–3.0 GHz data, without modification of intermediate initial guesses (Scenario A—**top**), using tissue-dependent mapping at fixed 200 iterations per frequency (Scenario B—**middle**), and employing stopping criteria and tissue-dependent mapping (Scenario C—**bottom**).

Having established an appropriate set of variables to govern the stopping criteria, a series of simulations adhering to the full description of the technique in Section 2.5 could be carried out to demonstrate its benefit in efficiency over conventional methods. The REN values of three scenarios (A, B, and C from Table 1) comparing fixed-iteration reconstructions (with and without tissue-dependent mapping) to the use of both tissue-dependent mapping and the stopping criteria, are shown in Figure 7. The final imaging reconstructions of these three cases are also depicted in Figure 8. As the proposed stopping conditions introduce an unpredictable halt to the reconstruction cycle that could occur at any frequency and also includes a mandatory return to the lowest frequency (in this case, 1.0 GHz) for the final optimization, each REN data point may not represent consecutive frequencies of the cycle. For consistency, the REN values shown in Figure 7 are therefore all calculated based on the same 2.0 GHz model from Figure 4. For clarity, the frequency associated with each data point plotted in Figure 7, along with the triggering condition that caused a change in frequency or termination in the reconstruction (when relevant), is shown in Table 1.

Although DGM-CSI, like other iterative microwave imaging algorithms, performs reasonably well in recovering the real component of breast models it reconstructs, it has difficulty producing an accurate profile of the imaginary part, most notably at higher frequencies. This deficiency is illustrated from the Scenario A inversion of the Category C breast model in the top image of Figure 8. Subjectively, the benefit of employing the tissue-dependent mapping in the fidelity of the imaginary component is obvious in both the middle and bottom images. It has been already shown elsewhere that the described mapping technique and “anchoring” of the imaginary component at each subsequent frequency stage, along with the practice of frequency cycling, imparts a benefit to the quality of the real part of the reconstructions following the same number of iterations [24,25]. However, these other studies only tested reconstructions that used fixed numbers of iterations as termination conditions; the addition of the stopping criteria for the latter case here (Scenario C) does not appreciably degrade the quality of the final image, while reducing the total number of iterations by over 35%.

### 3.2. Imaging with PEC Boundaries

**Figure 9:**
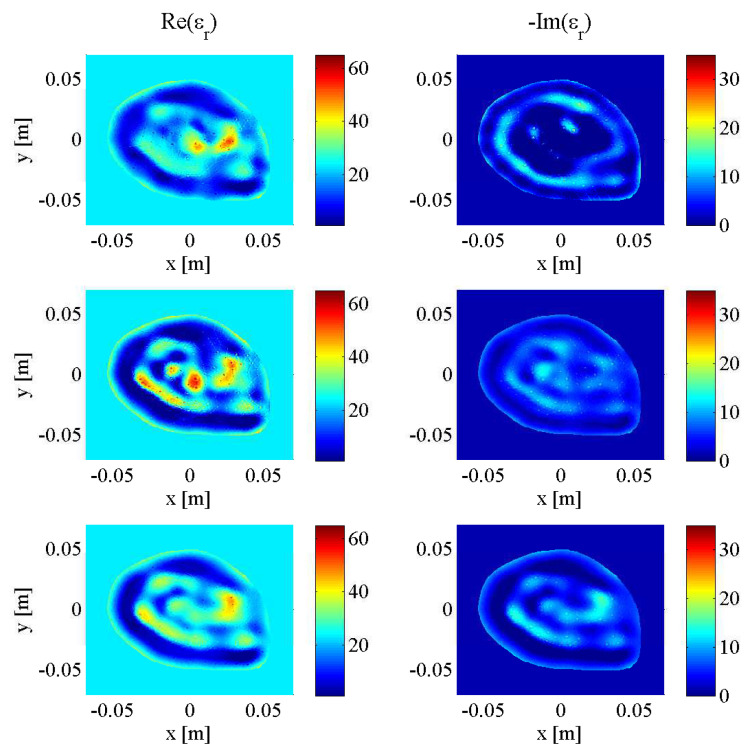
Final results of DGM-CSI frequency-hopping and frequency-cycled complex dielectric property reconstruction of PEC-bounded synthetic breast model using 1.0–1.5 GHz data, without modification of intermediate initial guesses (Scenario D—**top**), using tissue-dependent mapping at fixed 200 iterations per frequency (Scenario E—**middle**), and employing stopping criteria and tissue-dependent mapping (Scenario F—**bottom**).

**Figure 10:**
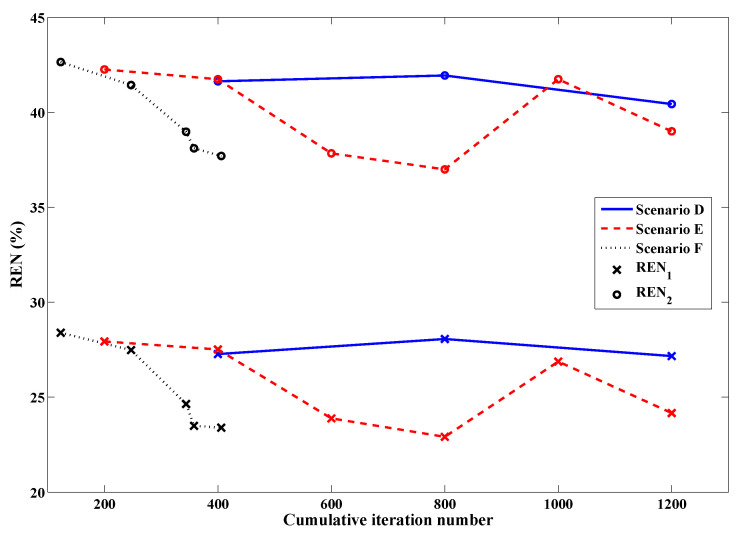
Relative error norms of reconstructions with PEC boundaries: Scenario D (solid line)—without tissue-dependent mapping at 400 iterations per frequency terminating after first inversion of 1.5 GHz data, Scenario E (dashed line)—with tissue-dependent mapping at 200 iterations per frequency, cycling through reconstruction frequencies once (with imaginary component “anchored” following initial 1.0 GHz inversion), Scenario F (dotted line)—with tissue-dependent mapping and stopping criteria in place, terminating after two consecutive inversions of 1.0 GHz data (one with the imaginary component “anchored” and the final run with the imaginary component freely optimized according to the guidelines of Section 2.5). See Table 2 for further details.

A set of simulations similar to those carried out in the previous section were performed using PEC boundaries and data from frequencies of 1.0 GHz, 1.25 GHz and 1.50 GHz, to demonstrate this method’s robustness to smaller frequency bandwidths and different boundary conditions, as mentioned in Section 2.6. Scenarios D and E are imaging simulations recreated from an earlier study that employed fixed-iteration reconstructions exclusively [25]; they are included in order to emphasize the benefit of adding stopping criteria in Scenario F. The imaging results are shown in Figure 9 with a breakdown of the reconstruction progression documented in Table 2. A plot of the relative error norms again similar to the open boundary cases, employing REN calculations based on the original 1.25 GHz model, is also provided in Figure 10.

As explained in [25], it was expected that the results would not be as impressive as open-boundary scenarios since PEC-bounded problems are generally more difficult to solve, and data was taken at lower frequencies. However, it is again clear from Figure 9 that there is an improvement in the recovery of the imaginary component when tissue-dependent mapping is used (middle and bottom images), more so for Scenario F. A modest improvement in the real part of $\varepsilon_{r}$ for Scenarios E and F is also noted. Without the use of stopping criteria, however, Scenario E overshoots the expected values of dielectric constant in several areas. Its REN values depicted in Figure 10 correspondingly suffer increases relating to the fact that the reconstruction cycle has perhaps been allowed to run too long, as pointed out in [25]. The introduction of the stopping criteria has effectively prevented this degradation by terminating the reconstruction cycle close to the observed nadir of these error values from Scenario E, while once again demonstrating a sizable increase in computational efficiency by reducing the number of iterations by an impressive 66%.

### 3.3. Effect of Noise Levels

**Figure 11:**
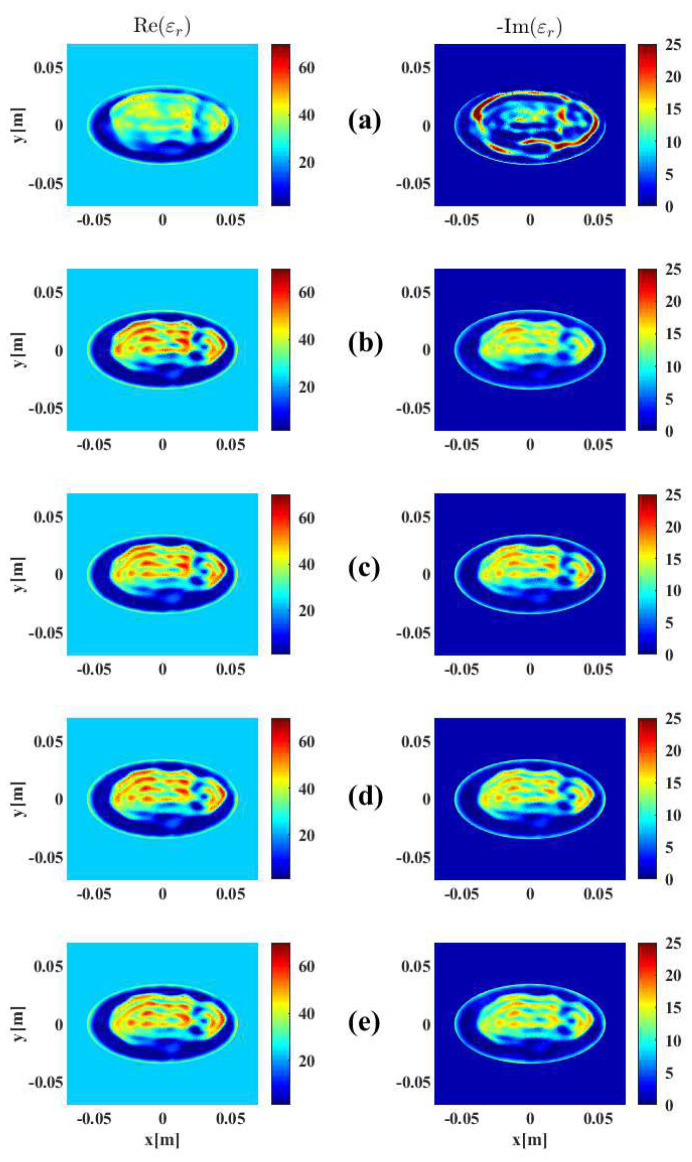
Final results of DGM-CSI frequency-hopping and frequency-cycled complex dielectric property reconstruction of open-boundary Category D synthetic breast model using 1.0–3.0 GHz data: (**a**) Scenario G—without tissue-dependent mapping at 400 iterations per frequency terminating after first inversion of 3.0 GHz (5% noise), (**b**) Scenario H—with tissue-dependent mapping and stopping criteria in place at 3% noise, (**c**) Scenario I—with tissue-dependent mapping and stopping criteria in place at 5% noise, (**d**) Scenario J—with tissue-dependent mapping and stopping criteria in place at 7.5% noise, (**e**) Scenario K—with tissue-dependent mapping and stopping criteria in place at 10% noise. See Table 3 for further details.

**Figure 12:**
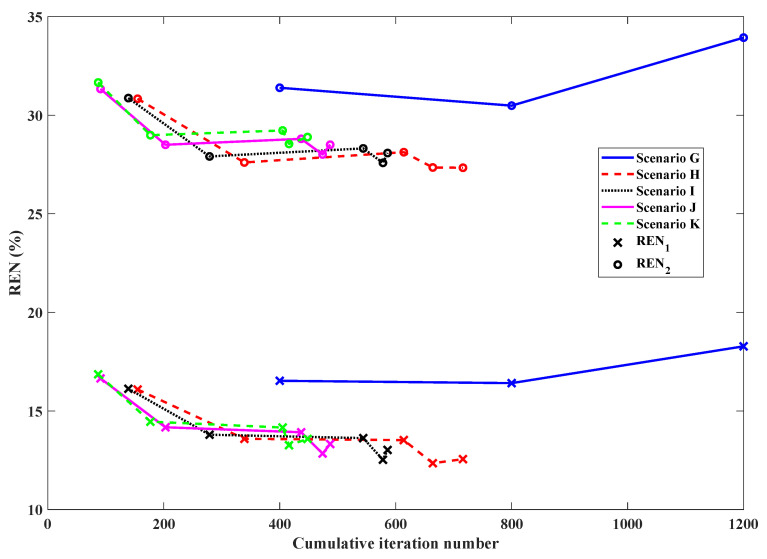
Relative error norms of frequency-hopping and frequency-cycled reconstructions of Category D model: Scenario G (blue solid line)—without tissue-dependent mapping at 400 iterations per frequency terminating after first inversion of 3.0 GHz (5% noise), Scenario H (red dashed line)—with tissue-dependent mapping and stopping criteria in place at 3% noise, Scenario I (black dotted line)—with tissue-dependent mapping and stopping criteria in place at 5% noise, Scenario J (magenta solid line)—with tissue-dependent mapping and stopping criteria in place at 7.5% noise, Scenario K (green dashed line)—with tissue-dependent mapping and stopping criteria in place at 10% noise. See Table 3 for further details.

To validate that the stopping criteria developed and tested on the BI-RADS Category C 2D model would perform similarly on different breast models and be robust to varying levels of noise, another full set of simulations were carried out on the aforementioned Category D model from the University of Wisconsin database using open boundary data at 1.0 GHz, 2.0 GHz, and 3.0 GHz (Figure 5). The uniformly-distributed noise used to corrupt the $E_{z}$ scattered electric field data was generated at 3%, 5%, 7.5%, and 10% of the maximum field magnitude. The imaging results are shown in Figure 11 with a breakdown of the reconstruction progression documented in Table 3. A plot of the relative error norms again similar to the Category C cases, employing REN calculations based on the original 2.0 GHz Category D model, is also provided in Figure 12.

As shown in the final images of Figure 11, there is an obvious improvement using tissue-dependent mapping and stopping criteria over the simple frequency-hopping approach (Scenario G), especially in the imaginary part, whose geometry is not reconstructed properly and contains a large overshooting boundary artifact. However, remarkably, there is very little subjective difference in the images reconstructed from data corrupted with different levels of noise (from 3% to 10% for Scenarios H through K), with subtle changes in the real and imaginary parts visible only with careful scrutiny.

The relative error norms of all scenarios depicted in Figure 12 and the reconstruction break-down in Table 3 offer more objective measurements of the effect of noise. Specifically, lower final REN values are observed for images inverted from data containing lower noise levels, as would be expected. Also, the stopping criteria transitions frequencies and halts the entire reconstruction cycle sooner for higher noise levels. These trends are consistent for all reconstructions employing tissue-dependent mapping and stopping criteria in each level of noise used in the simulations. For instance, data containing 3% noise (Scenario H) runs longest, for a total of 759 iterations; this total is observed to gradually decrease at the 5% and 7.5% noise levels, and data containing 10% noise (Scenario K) runs for only 448 iterations.

These observations are congruous with the theory introduced in Section 2.3 that data containing higher magnitudes of noise would have less useful information for the optimization algorithm to extract before the distribution of data error reached a steady state (i.e., before the algorithm began to reconstruct noise) and continuing the inversion would be superfluous, or perhaps even detrimental to the final image. Overall, while these additional 2D simulations do not necessarily represent a fully comprehensive investigation across all possible breast models and imaging scenarios, the performance of the proposed stopping criteria appears satisfactory on the Category D model, and behaves as expected on noisier datasets, even with parameter choices derived from the Category C model reconstructions.

## 4. Conclusions

It has been demonstrated that a stopping condition based on the similarities of the statistical distribution of data error at successive iterations has the capability of significantly speeding up the DGM-CSI algorithm while having no appreciable detrimental effect on the final imaging results, even yielding superior solutions than fixed-iteration scenarios. This boost of efficiency is granted through novel use of sequential two-sample K-S tests on the data error to determine appropriate times to halt the imaging algorithm, truncating likely unnecessary (and in some cases counterproductive) reconstruction iterations.

Whether or not the best set of parameters used in this analysis represents the true optimal values for the stopping criteria for other breast models, or if these same values are applicable to 3D inversions, is open to debate. However, given the promising imaging results obtained from this initial set of tests, and the gains in efficiency provided through significant reduction of the number of iterations needed to converge to these solutions, it was judged appropriate to keep the best parameters from this introductory exploration of the method and leave a more comprehensive examination of parameter optimization for future investigations.

The most interesting form of the proposed multi-frequency imaging procedure employs tissue-dependent mapping and “anchoring” of the imaginary part along with the new stopping criteria. This ad hoc procedure also returns the reconstruction cycle to stable low-frequency data after recovering anatomical detail from higher frequencies, allowing both real and imaginary parts to properly converge before global termination. The advantages gained by this use of both tissue-dependent mapping and novel stopping criteria have also been demonstrated to be robust for different breast models, frequency bandwidths, boundary conditions, and noise levels.

In fact, the stopping criteria have a more dramatic effect on the imaging results obtained from the more complex PEC-bounded problem and data with higher noise levels, suggesting this technique may be especially useful for avoiding image degradation and reconstruction artifacts in difficult cases such as full 3D reconstructions in semi-resonant enclosures, including experimental measurements that may be excessively noisy or contain poor frequency data unsuitable for inversion. To explore this possibility, preliminary extension of this technique to the 3D DGM-CSI algorithm developed at the University of Manitoba is a logical next step, along with parameter optimization studies of the stopping criteria for data from experimental systems. 

## Figures and Tables

**Table 1:** Reconstruction progression of open boundary scenarios (Figure 7).

			Frequency:						
Scenario	TM	SC	No. of Iterations (Stopping Condition)						Total
			*Components Reconstructed*						
			**1.0 GHz:**	**2.0 GHz:**	**3.0 GHz:**				
A	No	No	400 (F)	400 (F)	400 (F)				1200
			*Re, Im*	*Re, Im*	*Re, Im*				
			**1.0 GHz:**	**2.0 GHz:**	**3.0 GHz:**	**1.0 GHz:**	**2.0 GHz:**	**3.0 GHz:**	
B	Yes	No	200 (F)	200 (F)	200 (F)	200 (F)	200 (F)	200 (F)	1200
			*Re, Im*	*Re*	*Re*	*Re*	*Re*	*Re*	
			**1.0 GHz:**	**2.0 GHz:**	**3.0 GHz:**	**1.0 GHz:**	**2.0 GHz:**	**1.0 GHz:**	
C	Yes	Yes *	140 (KS)	190 (KS)	255 (KS)	53 (KS)	89 (DE)	41 (KS)	768
			*Re, Im*	*Re*	*Re*	*Re*	*Re*	*Re, Im*	

TM = tissue mapping; SC = stopping criteria; F = fixed; KS = Kolmogorov-Smirnov test window; DE = Domain error; *Re* = Real part; *Im* = Imaginary part. * 80% of a 30-iteration data error window must reach or surpass a K-S test *p*-value threshold of 0.99.

**Table 2:** Reconstruction progression of PEC-bounded scenarios (Figure 10).

			Frequency:						
Scenario	TM	SC	No. of Iterations (Stopping Condition)						Total
			*Components Reconstructed*						
			**1.0 GHz:**	**1.25 GHz:**	**1.5 GHz:**				
D	No	No	400 (F)	400 (F)	400 (F)				1200
			*Re, Im*	*Re, Im*	*Re, Im*				
			**1.0 GHz:**	**1.25 GHz:**	**1.5 GHz:**	**1.0 GHz:**	**1.25 GHz:**	**1.5 GHz:**	
E	Yes	No	200 (F)	200 (F)	200 (F)	200 (F)	200 (F)	200 (F)	1200
			*Re, Im*	*Re*	*Re*	*Re*	*Re*	*Re*	
			**1.0 GHz:**	**1.25 GHz:**	**1.5 GHz:**	**1.0 GHz:**	**1.0 GHz:**		
F	Yes	Yes *	123 (KS)	124 (KS)	97 (KS)	14 (DE)	48 (DE)		406
			*Re, Im*	*Re*	*Re*	*Re*	*Re, Im*		

TM = tissue mapping; SC = stopping criteria; F = fixed; KS = Kolmogorov-Smirnov test window; DE = Domain error; *Re* = Real part; *Im* = Imaginary part. * 80% of a 30-iteration data error window must reach or surpass a K-S test *p*-value threshold of 0.99.

**Table 3:** Reconstruction progression of Category D model noise-variant scenarios (Figure 12).

			Frequency:						
Scenario (Noise %)	TM	SC	No. of Iterations (Stopping Condition)						Total
			*Components Reconstructed*						
			**1.0 GHz:**	**2.0 GHz:**	**3.0 GHz:**				
G (5%)	No	No	400 (F)	400 (F)	400 (F)				1200
			*Re, Im*	*Re, Im*	*Re, Im*				
			**1.0 GHz:**	**2.0 GHz:**	**3.0 GHz:**	**1.0 GHz:**	**2.0 GHz:**	**1.0 GHz:**	
H (3%)	Yes	Yes *	155 (KS)	140 (KS)	265 (KS)	50 (KS)	52 (DE)	43 (DE)	759
			*Re, Im*	*Re*	*Re*	*Re*	*Re*	*Re, Im*	
			**1.0 GHz:**	**2.0 GHz:**	**3.0 GHz:**	**1.0 GHz:**	**1.0 GHz:**		
I (5%)	Yes	Yes *	139 (KS)	140 (KS)	265 (KS)	34 (DE)	8 (DE)		586
			*Re, Im*	*Re*	*Re*	*Re*	*Re, Im*		
			**1.0 GHz:**	**2.0 GHz:**	**3.0 GHz:**	**1.0 GHz:**	**1.0 GHz:**		
J (7.5%)	Yes	Yes *	91 (KS)	112 (KS)	234 (KS)	37 (DE)	13 (DE)		487
			*Re, Im*	*Re*	*Re*	*Re*	*Re, Im*		
			**1.0 GHz:**	**2.0 GHz:**	**3.0 GHz:**	**1.0 GHz:**	**1.0 GHz:**		
K (10%)	Yes	Yes *	87 (KS)	90 (KS)	228 (KS)	11 (DE)	32 (DE)		448
			*Re, Im*	*Re*	*Re*	*Re*	*Re, Im*		

TM = tissue mapping; SC = stopping criteria; F = fixed; KS = Kolmogorov-Smirnov test window; DE = Domain error; *Re* = Real part; *Im* = Imaginary part. * 80% of a 30-iteration data error window must reach or surpass a K-S test *p*-value threshold of 0.99.
